# Changes in the geographical distribution and abundance of the tick *Ixodes ricinus *during the past 30 years in Sweden

**DOI:** 10.1186/1756-3305-5-8

**Published:** 2012-01-10

**Authors:** Thomas GT Jaenson, David GE Jaenson, Lars Eisen, Erik Petersson, Elisabet Lindgren

**Affiliations:** 1 Medical Entomology Unit, Department of Systematic Biology, Evolutionary Biology Centre, Uppsala University, Norbyvägen 18d, SE-752 36 Uppsala, Sweden; 2 Department of Microbiology, Immunology and Pathology, Colorado State University, Fort Collins, CO 80526, USA; 3 Erik Petersson, Department of Animal Ecology, Evolutionary Biology Centre, Uppsala University, Norbyvägen 18d, SE-752 36 Uppsala, Sweden; 4 Elisabet Lindgren, Institute of Environmental Medicine, Karolinska Institutet, SE-171 77 Stockholm, Sweden

## Abstract

**Background:**

*Ixodes ricinus *is the main vector in Europe of human-pathogenic Lyme borreliosis (LB) spirochaetes, the tick-borne encephalitis virus (TBEV) and other pathogens of humans and domesticated mammals. The results of a previous 1994 questionnaire, directed at people living in Central and North Sweden (Svealand and Norrland) and aiming to gather information about tick exposure for humans and domestic animals, suggested that *Ixodes ricinus *ticks had become more widespread in Central Sweden and the southern part of North Sweden from the early 1980s to the early 1990s. To investigate whether the expansion of the tick's northern geographical range and the increasing abundance of ticks in Sweden were still occurring, in 2009 we performed a follow-up survey 16 years after the initial study.

**Methods:**

A questionnaire similar to the one used in the 1994 study was published in Swedish magazines aimed at dog owners, home owners, and hunters. The questionnaire was published together with a popular science article about the tick's biology and role as a pathogen vector in Sweden. The magazines were selected to get information from people familiar with ticks and who spend time in areas where ticks might be present.

**Results:**

Analyses of data from both surveys revealed that during the near 30-year period from the early 1980s to 2008, *I. ricinus *has expanded its distribution range northwards. In the early 1990s ticks were found in new areas along the northern coastline of the Baltic Sea, while in the 2009 study, ticks were reported for the first time from many locations in North Sweden. This included locations as far north as 66°N and places in the interior part of North Sweden. During this 16-year period the tick's range in Sweden was estimated to have increased by 9.9%. Most of the range expansion occurred in North Sweden (north of 60°N) where the tick's coverage area doubled from 12.5% in the early 1990s to 26.8% in 2008. Moreover, according to the respondents, the abundance of ticks had increased markedly in LB- and TBE-endemic areas in South (Götaland) and Central Sweden.

**Conclusions:**

The results suggest that *I. ricinus *has expanded its range in North Sweden and has become distinctly more abundant in Central and South Sweden during the last three decades. However, in the northern mountain region *I. ricinus *is still absent. The increased abundance of the tick can be explained by two main factors: First, the high availability of large numbers of important tick maintenance hosts, i.e., cervids, particularly roe deer (*Capreolus capreolus*) during the last three decades. Second, a warmer climate with milder winters and a prolonged growing season that permits greater survival and proliferation over a larger geographical area of both the tick itself and deer. High reproductive potential of roe deer, high tick infestation rate and the tendency of roe deer to disperse great distances may explain the range expansion of *I. ricinus *and particularly the appearance of new TBEV foci far away from old TBEV-endemic localities. The geographical presence of LB in Sweden corresponds to the distribution of *I. ricinus*. Thus, LB is now an emerging disease risk in many parts of North Sweden. Unless countermeasures are undertaken to keep the deer populations, particularly *C. capreolus *and *Dama dama*, at the relatively low levels that prevailed before the late 1970s - especially in and around urban areas where human population density is high - by e.g. reduced hunting of red fox (*Vulpes vulpes*) and lynx (*Lynx lynx*), the incidences of human LB and TBE are expected to continue to be high or even to increase in Sweden in coming decades.

## Background

The annual incidences in Europe of tick-borne diseases, particularly Lyme borreliosis (LB) and tick-borne encephalitis (TBE), have been increasing since the 1980s [1,2]. In Sweden, since 1988 every year except 1996 and 2010 has been warmer or much warmer than the long-term average for 1961-1990. Furthermore, between 1880 and 2009 the mean temperature in Sweden increased by 2°C [3]. This is in line with an increasing green-house effect [4]. During the same time period mean global temperatures increased and further warming is predicted [5]. This poses the question of whether or not there is a cause-and-effect relationship so that a warmer climate directly and/or indirectly contributes to increased tick abundance and increased intensity or geographical range of enzootic pathogen transmission leading to greater risk for human disease [6-9]. Biological phenomena or processes usually depend on many interacting factors. In some European countries climate change may have had only a marginal effect on the recent increase in human incidence of TBE; Instead, political and socio-economic alterations were presumably the main drivers causing increased contact between humans and infective ticks thereby augmenting the incidence of human TBE [10,11].

Before the early 1980s the northern range limit of the pathogen-transmitting tick, *I. ricinus*, was considered to be determined by the important biogeographical boundary called Limes Norrlandicus (LN), which runs through Central Sweden and separates the Nemoral zone to the south of the LN from the Boreal zone to the north [12]. The first comprehensive mapping of the geographical distribution of *I. ricinus *in Sweden was carried out in 1992-94 [13]. Moreover, in 1994 a questionnaire study was performed with the aim of exploring whether the northern limit of the tick had changed [14]. The new northern boundary for *I. ricinus *was shown to run through Central Sweden and along coastal North Sweden. During the same period the main host in Sweden for adults of *I. ricinus*, the roe deer (*Capreolus capreolus*) rapidly increased its population size: In 1955 there were about 100 000 roe deer in Sweden. In 1985 the population numbered about 300 000 deer when it began to increase more rapidly: in 1993-94 it was > 1 million deer [15,16]. Main reasons for this "population explosion" were an epizootic of scabies (*Sarcoptes scabiei*) in the two most important predators of roe deer, namely the red fox (*Vulpes vulpes*) and the lynx (*Lynx lynx*) [17,18] populations from early 1970's to late 1980's, and a series of mild winters during the early 1990's [16]. These factors reduced mortality and promoted survival and reproduction of roe deer. Both predator populations increased during the 1990's which, together with deer hunting, resulted in a decline of the roe deer population. The last census in 2005 estimated the roe deer population in Sweden to be about 375 000 deer [15,19] when more roe deer were shot than the total population numbered 50 years earlier. Since 2005 the roe deer population has declined further, especially during the two harsh winters, with deep snow cover, in 2009-10 and 2010-11 [15,19].

About 1,200 respondents, mainly from Central and North Sweden answered the short questionnaire that was published in the spring of 1994 in national free magazines for home owners, local newspapers and two major magazines for dog owners [14]. The questionnaire asked, among other things, if ticks were present in the vicinity of the respondent's residence during the previous two years (1992-1993) and in the early 1980s. The results suggested that, during this 10-year period, *I. ricinus *had spread to new localities north of its previous range; and across most of its previous northern range ticks had become more abundant. The answers to the questionnaire also suggested that there was a boundary zone across south-central Sweden where tick abundance changed from high in the southern part to low in the northern part of the zone [14]. In August-September 1994-96 the density of ticks was estimated by cloth-dragging at 57 localities in the hypothetical boundary zone between 60° 10' and 60° 55'N. The results of that field study confirmed that there was such a boundary zone; to the south of this zone host-seeking *I. ricinus *ticks could, in general, be found but not to the north of it except sparsely along the Baltic Sea coastline [14].

A similar picture has been documented in Central Europe: In the mountainous regions of the Czech Republic, *I. ricinus *is now present at higher altitudes than in the 1980s [20,21]. This changed tick distribution is associated with increased temperatures at higher altitudes [21]. In Denmark, tick density was found to be related to roe deer density [22] and the drastic increase in tick abundance in Denmark from 1985 to the beginning of the 1990s could be explained by increased temperatures and deer density [23]. A survey throughout Great Britain indicated that the opinion of most people was that there are more ticks today than in the past and that these increases in tick abundance coincided spatially with increases in deer numbers [24]. A recent analysis of various data on ticks and tick-borne pathogens collected over several decades in Norway indicated that *I. ricinus *is now found further to the north and at higher altitudes than a few decades ago [25].

Both the latitudinal changes in tick distribution and abundance in Sweden and the altitudinal changes in the Czech Republic have been found to be correlated with changes in temperatures and number of degree-days per season [7,20,26]. Since the 1990s changes in climate have continued [3,5] which may have further influenced the range and abundance of *I. ricinus *in Sweden. In addition, previous studies have shown that the risk of contracting LB in Sweden coincides with the distribution and density of *I. ricinus *[7,27-30]. In other words, changes in tick distribution and density are likely to have increased the risk of human LB and are likely to lead to further changes in risk areas for LB.

This study was a follow-up investigation 15 years after the first major national investigation of the distribution and abundance of *I. ricinus *in Sweden took place. Our main aim was to investigate if there had been any changes, since the 1994 study, in distribution and abundance of *I. ricinus *in Central and North Sweden.

## Methods

In April-June 2009 a short questionnaire (Appendix 1), almost identical to the one used in 1994, was attached to a popular science article on ticks and tick-borne diseases which was published in the free magazines Apoteket (The Pharmacy; available from May-August 2009 at all Swedish pharmacies) and Vi i Villa (We Home Owners; distributed to all Swedish home owners), as well as in major national journals for dog owners (Brukshunden; The Service Dog) and hunters (Svensk Jakt; Swedish Hunting), in four local North-Swedish newspapers (Länstidningen Östersund, Norran, Norrbottenskuriren, Piteåtidningen), and on a Swedish website http://www.blodsugare.se with information about ticks and tick-borne infections.

In the questionnaire of the 2009 study we asked for information about the occurrence of ticks within 1 km of the respondent's residence. We pointed out that we were particularly interested in answers from the northern and central regions of Sweden. If ticks were reported to be present, a follow-up question focused on approximately how many were found on each family member and on each one of the family's dog(s) and cat(s) in 2008. In the analyses of these data, responses of "many", "several" or "some" rather than a numerical value were excluded from the calculations of median numbers of ticks on tick-infested hosts (with at least one tick found per host during the tick season). The numbers of uninfested hosts were recorded separate from the infested hosts to permit calculations of percentages of hosts without any tick(s) recorded on their bodies. We also asked if ticks were present in the same area in the beginning of the 1990s, and if the respondent considers ticks to have become more or less prevalent since that time period, or if no obvious change in tick abundance had occurred. We specifically stated that we were equally interested in "No" answers as in "Yes" or "No change" answers. We also asked for ticks (removed from humans and domestic animals, including information about date of collection, locality and host species) to be sent for species identification to one of the authors (TJ). The identification of these ticks is in progress and will be presented in a separate publication. However, in Sweden nearly all (> 99%) ticks found on humans, dogs, cats, horses, cattle, hares and deer are *I. ricinus *[13]. We therefore consider the data presented in this paper to refer to *I. ricinus*. The word "tick", when used in this article, denotes *I. ricinus*.

In the Results section we use the term "distant memory answers" to denote answers that indicate that the respondent tries to remember the "tick situation" > 10 years ago. The term "actual, near-present tick situation" is used to indicate that the respondent attempts to remember the "tick situation" 1-2 years ago.

### Critique of the questionnaire-based method used

The ideal way to detect changes in the geographic range and density of *I. ricinus *would be to sample for ticks regularly at many locations throughout the country, even at locations where the tick is presently not known to occur. Such a tick monitoring programme would be exceedingly time-consuming and expensive and, therefore, has not and is not carried out in Sweden. In the absence of such a surveillance programme we consider that this investigation, based on questionnaires, is an appropriate alternative. However, we want to emphasize that some factors reduce the reliability of the data collected. Positive results are likely to be reported more frequently than negative ones. Therefore, data may be biased towards locations where ticks were present, where ticks had increased in abundance or where they had recently become established [24]. People are nowadays likely to be much more aware of what a tick looks like and more alert to detect ticks compared to in the 1960s-70s, i.e., before Lyme borreliosis had been described. Also, people generally remember more correctly something that happened recently compared to things that happened long ago. These phenomena presumably influenced the data. Furthermore, people interested in tick biology, persons who have contracted a tick-borne disease and people who have many ticks in their garden, on their cats or dogs, etc. are more likely than others to answer the questionnaire. In the questionnaire, we stated that we were equally interested in receiving "negative" answers, i.e. no ticks present, as "positive" answers. Thus, another potentially biasing factor is that people, particularly in North Sweden where ticks had rarely been observed before ca. 1990, who recently had discovered ticks near their homes were presumably more prone to respond more readily to the questionnaire than people who had never observed any ticks near their homes.

### Data analyses

**Figure 1:**
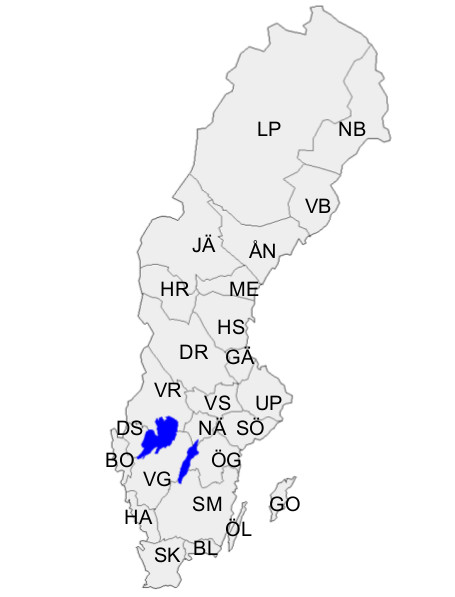
**Swedish provinces (landskap) and their abbreviations**.

Only questionnaires (N = 1,032) that provided acceptable, i.e. complete answers for both time periods (early 1990s and 2008) were included in the analyses. Data on tick occurrence (presence/absence) in the different regions were calculated using generalized linear methods [31], binomial distribution, i.e. logistic regression; region being the class variable. The response variable being number of adequately filled in questionnaire forms reporting ticks out of the total number of correctly filled in questionnaires for each province (see Figure 1). If an overall difference between regions was indicated we performed a post-hoc pair-wise test for the probability of tick presence. In this case we used a Wald χ^2^. The Wald test [32] is based on the idea that we accept the null hypothesis when the observed θ is close to θ_0_. The distance between observed θ and θ_0 _is the basis of constructing the test statistics. Typically the square of the difference is compared to a χ2-distribution [33].

The data for tick presence on man, dog or cat could not be analyzed with general linear methods. Instead an ordinary ANOVA was performed, and the pair-wise comparisons among regions were done using the t-test. Data for other pair-wise comparisons, e.g. change in tick abundance before vs. after a certain time, exhibited a non-normal distribution. In these cases, we used Wilcoxon's matched-pair signed-ranks test. To test for differences in change between regions or provinces the Kruskal-Wallis test was used. If an overall difference among regions or provinces was found a non-parametric pair-wise comparison method was used (Dunn's test). For most statistical analyses we used SAS 9.2 statistical software. However, Wilcoxon's matched-pair signed-ranks test was calculated by hand according to Siegel & Castellan [34] and the non-parametric pair-wise comparisons (Dunn's' test) were calculated according to Hollander & Wolfe [35].

**Figure 2:**
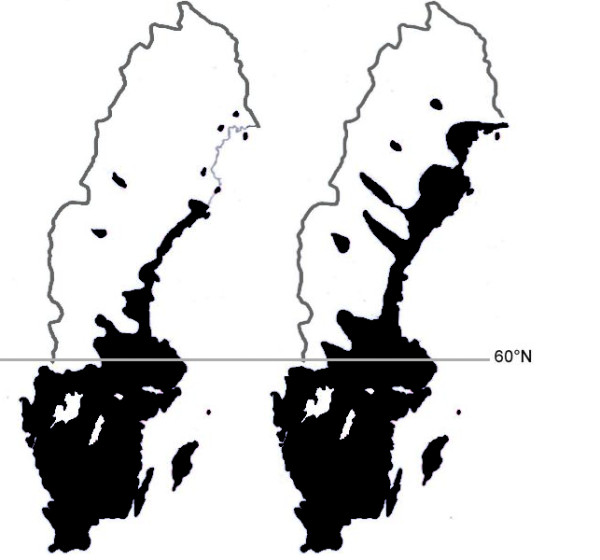
**Estimated ranges of *I. ricinus *in Sweden in the early 1990s (left map) and 2008 (right map)**.

**Figure 3:**
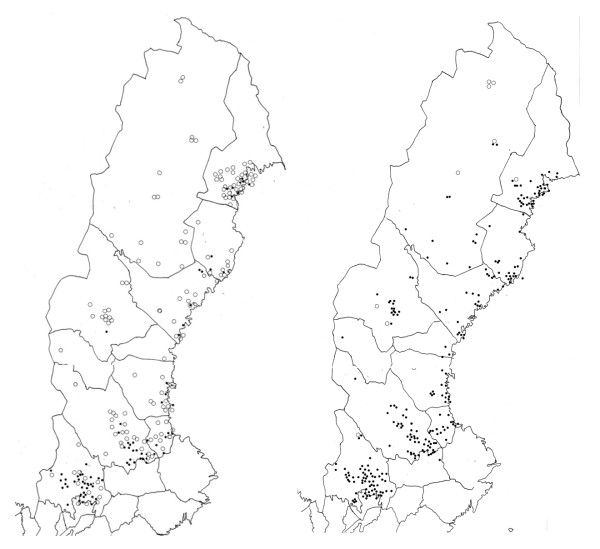
**Maps showing localities in Central and North Sweden where ticks (*I. ricinus*) were reportedly present (•) or absent (o) according to the respondents to a national tick survey in 2009**. Left map refers to the early 1990s and right map to 2008.

A computer program, written in PHP 5.4 beta 1 [36] was used to measure the number of pixels (where one pixel corresponds to 2.64 × 2.64 km) showing the estimated ranges of *I. ricinus *in the early 1990s and 2008 (Figure 2). The area where the tick was present in the early 1990s (Figure 2, left, black area) is based on Figure 3 (left map and Table 1) and a map with confirmed records of *I. ricinus *ticks identified in the laboratory (published in [13]). The map for 2008 (Figure 2, right, black) is based on the 2009 questionnaire reports of tick presence shown in Figure 3 (right map) and Table 1. By subtracting the number of pixels of the black area in the left map (Figure 2) from the number of pixels in black area of the right map (Figure 2) we obtained an estimate, expressed as a percentage, of how much the tick's range had changed from the early 1990s to 2008.

Data recorded in this study of 2009 was compared with data collected in 1994 covering the period from the early 1980s to 1993 and published by Tälleklint and Jaenson [14]. In the questionnaires of 1994 and 2009 we used the terms "early 1980s" and "early 1990s", respectively. We estimated these terms to signify ~1983 and ~1993, respectively. Thus, the first study period represents the years ~1983-1993 = ~11 years and the second period ~1993-2008 = ~16 years.

Names of the Swedish provinces (landskap) are abbreviated to two capital letters in accordance with the recommendations of the Swedish Entomological Society (Figure 1).

## Results

### Geographical distribution and abundance of *I. ricinus *ticks

Out of the 1,121 completed questionnaires returned by respondents to us, 1,032 questionnaires included complete answers for both time periods (early 1990s and 2008) and thus were deemed acceptable for inclusion in data analyses. During the early 1990s ticks were reported to be present in the vicinity of the homes of most respondents living in South Sweden (i.e. the provinces SK, BL, ÖL, GO, HA, VG, SM, ÖG, BO; 67.0% of replies indicating tick presence) and Central Sweden (DS, VR, NÄ, VS, SÖ, UP; 68.6% of replies indicating tick presence) (Table 1; Figure 3). By 2008, presence of ticks was reported even more commonly by respondents in South Sweden (99.1%) and Central Sweden (99.7%) (Table 1, Figure 3). Thus, by 2008 ticks were apparently present in practically all areas of South and Central Sweden. The data from North Sweden indicated dramatic increases in tick presence from the early 1990s to 2008. This included statistically significant increases from 33.3 to 97.4% for the southern part of North Sweden (DR, GÄ, HS); from 14.8 to 90.2% in the central part of North Sweden (ME, ÅN, HR, JÄ, VB); and from 9.5 to 73.0% for the two northernmost provinces in North Sweden (NB, LP).

**Table 1:** Results of two questionnaire studies, carried out in 1994 [14] and 2009 (this study), referring to changes in tick presence/absence and changes in tick abundance in different geographical regions of Sweden.

	Survey of 1994 **Data from questions asked during 1994 and published in [**14].				Survey of 2009 **Data from questions asked during 2009**.				
**Region (and provinces abbreviated)**	**Ticks present** **early 1980s** **n/N**	**Ticks present** **1993** **n/N**	**Change, %**	**No. respondents** **(N)**	**Ticks present early 1990s** **n/N**	**Ticks present 2008** **n/N**	**Change**, **%**	**"Ticks have become more numerous since early 1990s"** **% yes**	**No**. **respondents** **(N)**

**Northern North Sweden** (NB, LP)	4.9% 2/41	26.8% 11/41	+22^a^ (0.168)	41	9.5% 6/63	73.0% 46/63	+63^b^ (0.021)	71.4^a^ (< 0.001)	63

**Central North Sweden** (HR, ME,ÅN, JÄ, VB)	10.9% 17/156	42.3% 66/156	+31^b^ (0.004)	156	14.8% 9/61	90.2% 55/61	+75^b^ (0.004)	86.9^b^ (< 0.001)	61

**Southern North Sweden** (DR, GÄ, HS)	33.8% 93/275	72.4% 199/275	+39^b^ (0.058)	275	33.3% 26/78	97.4% 76/78	+64^b^ (0.005)	93.6^b^ (< 0.001)	78

**Central Sweden** (DS, VR, VS, NÄ, SÖ, UP)	77.4% 564/729	98.1% 715/729	+21^a^ (0.010)	729	68.6% 253/369	99.7% 368/369	+31^a^ (0.003)	91.3^b^ (< 0.001)	369

**South Sweden: West Coast** (HA, VG, BO)	N.D.	N.D.	N.D.	N.D.	64.5% 122/189	98.9% 187/189	+34^a^ (0.009)	88.8^b^ (< 0.001)	189

**South Sweden: Baltic islands **(ÖL, GO)	N.D.	N.D.	N.D.	N.D.	80.0% 20/25	100.0% 25/25	+20^a^ (0.052))	68.0^a^ (< 0.079)	25

**Southern South Sweden** (SK, BL, SM,ÖG)	N.D.	N.D.	N.D.	N.D.	67.6% 167/247	99.2% 245/247	+32^a^ (0.024)	86.2^b^ (< 0.001)	247

All regions *1201: only the 4 northernmost regions, i.e. Central, S. North, C. North and N. North	__	__	+29 (0.001)	1201*	58.4% 603/1032	97.1% 1002/103	+39 (0.001)	87.7	1,032

**n **= number respondents who answered "yes, ticks are/were present within 1 km from where I live" at that particular time (year), e.g. early 1980s or 1993 (= study of 1994), or early 1990s or 2008 = this study of 2009). **N **= total number of respondents in the region who answered the questionnaire. Values for **% change **appear in one column for each time period (early 1980s-1993 and early 1990s-2008); % change values in a column and denoted with the same letter (a or b) are not significantly different at the 5% level according to generalized linear model (binomial distribution). The value in parentheses under each value of % change shows the level of significance of the % change according to paired t-test. **N.D**. = not done. The next to last column shows the percentage of respondents, for each region in the study of 2009, who stated that "ticks had increased in numbers" (i.e. tick abundance had increased) during the last 16 years, in the respondents' residential areas. Thus, the percentages, in the next to last column, refer to % of respondents that replied "more numerous" to the question "Have ticks become more numerous, less numerous or have they not changed their abundance since the early 1990s in the area where you live?" *Not "All regions" The data on "% yes" were analyzed by the same statistical methods as the "% change" data

All Swedish tick records known to us were compiled during 1992-94 and published in [13]. Data from our first tick questionnaire study in 1994 were published in 1998 [14]. Both investigations showed that *I. ricinus *was present in all North-Swedish provinces except Härjedalen (HR). A similar pattern, i.e., with presence records of *I. ricinus *from all Swedish provinces except HR remained when the data of the 2009 questionnnaire study (this investigation) were analyzed. However, very recently - in August 2011 - we received on two separate occasions a total of four adult blood-fed tick females removed from two dogs treated at the District Veterinary Clinic at Hede, Härjedalen. These ticks were microscopically identified as *I. ricinus *and are now the first published finding of this species from Härjedalen. The dogs had not been away from the province during four weeks prior to detection of the ticks.

The results referring to 2008 (Figure 3) differ from the situation observed in the early 1990s in Central and North Sweden when ticks were, in general, only present close to the Baltic Sea Coast and only rarely encountered in the interior areas (Figure 2, based on a map previously published in Jaenson et al. [13]). This corroborates the impression of the maps in Figure 3 that from the early 1990s to 2008 the range of *I. ricinus *increased, particularly in North Sweden. Further to the south, ticks were already present in most locations investigated in the early 1990s (Figure 3 in [13]; Figure 2).

The estimated ranges of *I. ricinus *in Sweden in the early 1990s and 2008 are shown in Figure 2. Based on the number of tick presence pixels in each of the two maps, calculations revealed that the tick's distributional area in Sweden increased by 9.9% from early 1990s (41.6%) to 2008 (51.5%). Most of this range expansion occurred in North Sweden along the Baltic Sea coast. For North Sweden (north of 60°N) the tick's range increased by 14.3%, doubling from early 1990s (12.5%) to 2008 (26.8%).

### Comparison of tick occurrence between the study of 1994 and the present study

We compared data on tick occurrence for the two periods, i.e., the early 1980s - 1993 (i.e., questionnaire data recorded in 1994 and published in [14]) *versus *the early 1990s - 2008 (the present study of 2009). The "% change" in Table 1 refers to the percentage of respondents that considered ticks to be present within 1 km from the respondent's home. Statistical analysis suggests that the increase in "% change" in the three northern regions combined during the early 1990s - 2008 was greater than the "% change" during the early 1980s-1993 (Wald χ^2 ^= 16.32, p < 0001). In this analysis we took into account that the first period (early 1980s - 1993) is 11 years and the second period (early 1990s - 2008) 16 years. There was no interaction between region (Southern North, Central North, and Northern North Sweden) and time period (Wald χ^2 ^= 0.6417, P = 0.726). This suggests that the percentage increase was similar in the three northern regions.

### Change in tick distribution and abundance from early 1990s to 2008

The previous results refer to presence or absence of ticks within 1 km of the respondent's home. In 2009 we also asked if respondents considered tick numbers, i.e., tick abundance to have changed between the early 1990s and 2008. The next last column of Table 1 shows that in all regions, except the Baltic Islands, a significant proportion of the respondents considered that ticks had become more common (i.e., more numerous) since the early 1990s. The increased tick abundance was regarded as lower in the regions Baltic Islands and Northern North Sweden compared to the other regions.

In South and Central Sweden, where ticks were reported to have been present in 64.5-80% of the locations in the early 1990s, nearly all (98.9-100.0%) respondents stated that ticks "now" (2008) occur in the vicinity of their homes (Table 1). For North Sweden, there is a distinct increase reported in a number of locations where ticks are currently (2008) present compared to 16 years earlier. For example, in Central North Sweden respondents stated ticks to be present in 14.8% of locations in the early 1990s compared to 90.2% in 2008. For northernmost Sweden the corresponding proportions were 9.5% in early 1990s and 73% in 2008 (Table 1). In general the respondents' answers about presence of ticks gave greater values in 2008 than in 1994 (z = 1.761, p = 0.0392; Wilcoxon's matched-pair signed-rank test). The change in tick presence between 1993 and 2008 differed between the regions (χ^2 ^= 12.22, p = 0.016, Kruskal-Wallis test). Dunn's test for pair-wise comparisons revealed that the % change in tick presence from early 1990s to 2008 was greatest in the three regions of North Sweden and differed significantly from those of the Central and Southern regions (Table 1). During the same period, there was no significant difference in % change among the regions of North Sweden (Table 1). Similarly, no significant difference was found in % change among the four southern regions (Central, West Coast, Baltic Islands and southernmost Sweden (Table 1).

### The questionnaire results are affected by fading memory with time

The "distant memory-based tick presence" questionnaire data referring to the early 1990s to 2008 and collected in the study of 2009 were compared to the estimates of tick presence based on "near-present memory" for 1993 (study of 1994) and 2008 (study of 2009). The estimated change, increase in this case, according to respondents' "distant (faded) memory" was that tick presence increased on average 3.0 ± 2.9 times for the whole of Sweden, but according to "actual near-present" memory the estimated change was only 1.3 ± 0.5 times (t = 2.64, d.f. = 23.39, p = 0.0145, Satterthwaite's methods for unequal variance). Thus, the increase was greater according to "distant memory" compared to "near-present, actual" estimates. This likely reflects that the memory of the respondents becomes less reliable with time.

### Number of ticks per infested host

The estimated median numbers of ticks collected by the respondents on themselves and on their dogs or cats during the tick season of 2008 are shown for the different regions in Table 2. The median numbers of ticks recorded per respondent ranged between 4 and 7 in South and Central Sweden but were significantly lower (1-2) in North Sweden. For cats there is a tendency that tick numbers were greater in Central and South Sweden (20-30) than in North Sweden (2-11; Table 2). A similar tendency, although not statistically significant, was indicated by the median numbers of ticks on dogs: 12.5-25 for South and Central Sweden compared to 2-10 for North Sweden (Table 2). The proportion of uninfested hosts increased from southern Sweden towards the north (Table 2). This likely reflects the lower tick abundance in the north.

**Table 2:** Median number (in bold) of ticks per tick-infested host, i.e., respondent (*Homo sapiens*) and respondents' dogs (*Canis lupus familiaris*) and domestic cats (*Felis catus*) recorded during the "tick season" of 2008 for the different Swedish regions

Region (two or more provinces, "landskap")	Median, (no. obs.), maximum no. ticks per tick-infested human, and (no. and % of uninfested humans)	Median, (no. obs.), maximum no. ticks per tick-infested dog, and (no. and % of uninfested dogs)	Median, (no. obs.), maximum total no. ticks per cat, and (no. and % of uninfested cats)
**Northern North:** Norrbotten and Lappland	**1**^b ^NS (5) 1 (95; 95.0%)	**2**^a ^NS (26) 12 (5; 16.1%)	**1 ^ab ^**NS (34) 10 (4; 10.5%)
**Central North: **Medelpad and Ångermanland, Härjedalen, Jämtland, Västerbotten	**1.5**^a ^NS (2) 2 (22; 91.0%)	**2 ^a ^**NS (11) 20 (1; 8.6%)	**2 ^b ^**NS (10) 8 (1; 10.0%)
**Southern North: **Dalarna, Gästrikland and Hälsingland	**2**^a ^NS (43) 15 (35; 81.1%)	10 ^a ^** (45) 1000 (0; 0%)	**11**^b ^*** (36) 200 (1; 2.7%)
**Central: **Dalsland, Värmland, Västmanland, Närke, Södermanland and Uppland	5 ^b ^*** (311) 225 (58; 18.6%)	25 ^a ^*** (123) 1000 (0; 0%)	**27.5**^c ^*** (156) 100 (0; 0%)
**West Coast: **Halland, Västergötland and Bohuslän	**7**^b ^*** (163) 125 (26; 15.9%)	**18**^a ^*** (54) 500 (2; 3.5%)	**25**^bc ^*** (75) 1000 (1; 1.3%)
**South-East, Baltic Islands: **Öland and Gotland,	4 ^b ^*** (20) 52 (5; 20.0%)	**12.5**^a ^*** (6) 120 (1; 14.2%)	**20**^bc ^*** (10) 500 (0; 0%)
**South: **Skåne, Blekinge, Småland and Östergötland	**5 **^b ^*** (201) 125 (46; 18.6%)	**21.5**^a ^*** (86) 1500 (2; 2.3%)	**30**^b ^***(99) 250 (1; 1.0%)
**All provinces (landskap)**	5*** (745) 225 (287; 27.8%)	**15 **(351) 1000 (11; 3.0%)	**20 **(420) 1000 (8; 1.8%)

Medians denoted with the same letter in a column are not significantly different at p = 0.05 (t-test of geometric means). The asterisks at the Wald χ^2 ^-values indicate whether the corresponding geometric mean for the region is different from 0.0; ** 0.01 < P > 0.001, *** = P < 0.001, NS = not significant. In parentheses, after the median number, is shown the number of valid observations on which the median is based, and thereafter the maximum total number of tick specimens estimated by a respondent on one individual host. The last figures (in parentheses) in each column are the number and percentage of hosts without any ticks.

## Discussion

### Change in tick range and tick abundance

The results from both 1994 and 2008 surveys suggest that the range of *I. ricinus *increased markedly in Sweden during the period from the early 1980s to 2008. This range expansion appears quite distinct at the northern parts of the tick's range. Thus, *I. ricinus *now occurs in many localities in the interior and northernmost regions of Norrland in places where it was not present about three decades ago, albeit at lower densities compared to South and Central Sweden and the southern part of North Sweden. It should be noted that in contrast to South Sweden homesteads are to a greater extent located in the climatologically most suitable places which are along the coast and rivers in inland river valleys, around lakes and other "low-land" areas as far as possible protected from the cold northern climate. Therefore, the records of *I. ricinus *in Northern Sweden mainly refer to such clusters of respondents living in places with a milder climate, with a more "southern" vegetation and fauna than that of the surrounding boreal areas dominated by spruce and pine (taiga) forest.

Both questionnaire-based studies showed that there was a significant increase in the proportion of respondents that considered that "ticks are presently" (referring to the previous year's tick season) occurring near the respondents' homes, compared to 11-16 years ago. Comparison of the percentage increase in tick occurrence was greater, i.e., significantly more rapid during the second questionnaire study period (1993-2008) than during the first period (1983-1993). The winters of 1988 to 1995 were warm or exceptionally warm in Sweden [3]. As mentioned, this corresponds to the period when, due to the mild winters and a reduced fox population the number of roe deer increased dramatically to reach more than 1 million deer in 1994. The subsequent increase in fox and lynx numbers together with hunting began to reduce the roe deer population.

In view of the extended life cycle duration of *I. ricinus *which may be as long as 6 years in Northern Europe [37], there is a time lag of several years between a peak in the deer population and the resulting high *I. ricinus *population [38]. Thus, it is inferred that the greater "% change" per year of tick occurrence during the second study period (1993-2008) compared to that of the first period (1983-1993) is due to two factors: First, the high availability of large maintenance hosts, i.e. mainly roe deer, in particular during the late 1980s and early part of the 1990s should have been favourable especially for the adult ticks to reproduce and thereby for the tick population to increase. This conclusion is supported by data recorded at the Danish Pest Infestation Laboratory (DPIL) [23]: The annual numbers of requests for information on *I. ricinus *- a proxy for tick abundance - to DPIL was fairly stable between 1965 to 1985, but doubled during the late 1980s to reach a higher level in the early 1990s. The perceived tick abundance correlated with estimates of annual deer abundance and temperature records [23].

Second, for most European meteorological stations' temperature records the mean annual temperature shows a marked step-increase around 1989 and has thereafter been followed by consistently warm conditions [4,38]. Such an increased warming, most of which occurred from January to early August [38] should both directly and indirectly have favoured survival and reproduction of both roe deer and ticks [7]. Indirectly, climatic changes can significantly influence vegetation communities and tick host populations. Recent studies have shown a close correspondence between the durations of the vegetation period and snow cover period and the distributional area for *I. ricinus *in Sweden [7,39]. The tick is unlikely to become established in an area where the snow cover period is > 150 days/year and where the mean temperature is < 5°C for > 170 days. This suggests that a direct or indirect effect of the climate, i.e., temperatures within a certain range, determine the potential geographical distribution of *I. ricinus *- given that precipitation and humidity are adequate.

Like many other vectors of zoonotic pathogens [40]*I. ricinus *is a host- and habitat-generalist with a very wide host range and has been recorded from many different biotopes. The survival and proliferation of such ectoparasites are obviously dependent, in part, on the community of host species which they infest [40]. In general, the main hosts of adult *I. ricinus *on the Swedish mainland are cervids [13]. If, during a period of several years the climate is above "average favourable" for these mammalian tick hosts, they would presumably expand their population sizes and ranges - on the assumption that disease, predation and hunting pressure do not increase and that other tick hosts do not change their impact on the tick population. A corollary would be that *I. ricinus*, in the same area where the cervids occur, would most likely increase its geographical range and become more abundant. This is most likely what happened on the Swedish mainland during the last 30 years, in particular since 1988 to present time.

### Population dynamic aspects

In response to climate change, populations can shift their distribution, adapt to the new climate or go extinct; shifts of their distributions can be pole ward to higher latitudes or upwards to higher altitudes [41]. "Theory and limited empirical data suggest that shifts in population abundance along the edge of the range should be one of the first and most sensitive signs of a broader species response to environmental change" [41]. The range extension of *I. ricinus *in Sweden is at the northern edge of the tick's geographical distribution. It is in such marginal areas that we obtained the first indications about a response of *I. ricinus *to the changing climate and host abundance [14]. Here, at the limit of its range, environmental conditions are, in general, less optimal. The tick population will therefore be more likely to go extinct here than in its core area. Thus, it is likely that the distributional area of *I. ricinus *in North Sweden, as presented on the right map of Figure 3, might change: during some years the tick's range may enlarge whereas during other less favourable years it may retract.

Organisms are usually more abundant at the centre of their range, where optimal biotic and abiotic conditions usually prevail, than towards the periphery [42]. The data on median numbers of ticks recorded by the respondents on themselves, and on their dogs and cats suggest that these medians are greater in southern and central Sweden than in northernmost Sweden. This supports the view that tick density is, in general, much lower near the periphery of its range.

Near the core of the distributional range of *I. ricinus *in Central Europe a phenomenon similar to that observed by us in North Sweden was observed: In the Czech Republic *I. ricinus *extended its range to higher altitudes. Thus, ticks infected with the TBE virus (TBEV) and several species of the *B. burgdorferi *s.l. complex were collected during the last decade at higher altitudes than before [20,21,43]. Tick range expansion can be facilitated by human activity, for example when tick-infested dogs, cattle or other domesticated mammals are brought into previously tick-free areas. A few such cases where it is suspected that ticks, found in previously tick-free localities in northern Sweden, had dropped from dogs that had shortly before visited tick-infested areas in central or south Sweden were reported by informants during our survey (TGT Jaenson DGE Jaenson, unpublished data). However, we believe that in Sweden the main vehicle for rapid and effective transportation of ticks of all stages into new areas are roe deer and to a lesser extent other large mammals. Birds are important transporters of immature *I. ricinus*, which may be infected with *Borrelia *bacteria and other pathogens of humans [44-46]. In contrast to some other bird-parasitizing tick species, *I. ricinus *ticks on birds are very rarely adults [44-46]. Therefore, they are unlikely to establish new tick populations in tick-free areas. However, such immature ticks may introduce "new" pathogens into previously non-endemic areas.

### Roe deer - the main host for females of *Ixodes ricinus*

The abundance of ticks is largely determined by the availability of suitable hosts [22,23,29,30,47-49]. In many parts of Europe including Sweden, the roe deer has for the last decades up to the present time been the most important blood meal host for females of *I. ricinus *[13,14,22,23,48] and a mate-seeking site for the tick males [13]. Consequently, the roe deer population is a key factor for the reproductive success of the tick population. In North America, the white-tailed deer (*Odocoileus virginianus*) plays a similar role as an important host for *I. scapularis *[42,47,50]. In Central Sweden in the late summer, one single roe deer can harbour > 2000 *I. ricinus *ticks; Mean infestation rates of 30 females, 17 males, 93 nymphs and 265 larvae of *I. ricinus *were recorded on 37 roe deer by Tälleklint & Jaenson [51]. A growing number of roe deer is therefore likely to have been of major importance for the ticks increasing abundance and range expansion from the 1980s.

To obtain support for the hypothesis that there is a causal link between changes in deer distribution and abundance and similar changes in tick distribution and abundance, one would need to establish if such changes indeed have coincided in space and time [24]. In Great Britain people consider that the increases in tick numbers over recent years coincide spatially with increases in deer numbers; people's perceptions were supported by data showing simultaneous increases in tick infestation rates on grouse and roe deer [24].

For reasons explained earlier, the roe deer population in Sweden expanded to very high levels during the 1980s and 1990s, reaching a peak in 1993-94. The subsequent spread of ticks northwards in Sweden were most likely a result of the greater availability of hosts, particularly roe deer, and a changing climate that was directly and indirectly favourable for both ticks and roe deer [8,14,26]. Extended vegetation periods and mild winters have continued in most years after the early 1990s.

On the Swedish mainland the number of roe deer has recently declined, partly due to increasing numbers of predators but more importantly due to two cold winters (2009/2010 and 2010/2011) with heavy snow cover [15]. However, the roe deer has a great reproductive potential and great dispersal capacity [16], and during winter-time many hunters are providing fodder to increase deer survival and reproduction in order to maintain large numbers of deer for hunting. Therefore, if counter-measures are not undertaken, to keep the roe deer and other deer populations at low, acceptable levels in line with public health objectives, they are likely to rapidly regain high population levels.

The geographic range of roe deer in Sweden covers a larger area than the one where *I. ricinus *has been recorded. Thus, in 1990 roe deer were only absent from the north-western part of Lapland in North Sweden [16]. This implies that, provided the climate will be suitable for *I. ricinus *this tick may be able to establish permanent populations in areas of North Sweden where the tick is still absent but where the main host for the adult stage, the roe deer occurs.

A characteristic behavioural trait of young roe deer is their tendency to rapidly disperse to new areas, often far away from their place of birth [16]. Nearly all young deer in North Sweden appear to leave their place of birth [16]. While young deer in South Sweden usually do not disperse more than 20 km from their place of birth the corresponding mean distance in North Sweden is > 40 km with some of the migrations as far as 200 km [16], presumably due to greater distances between favourable habitats in North Sweden than in South Sweden. This behaviour strongly supports the hypothesis that roe deer has greatly contributed to the recent rapid and massive spread of *I. ricinus *throughout northern Sweden.

### What other factors are causing the increasing range and abundance of the tick and increased human incidence of tick-borne pathogens?

Changes in climate and vegetation are two key factors that will profoundly impact both *I. ricinus *and its host animals. Many other factors may be regarded as additional drivers that have affected and will affect the abundance and range of *I. ricinus *in Sweden and neighbouring countries. These include increased availability of large blood hosts, especially cervids, for the tick females and for both sexes to use as mating sites; migration and dispersal capacity of wild animals, especially deer; and migration and transportation of medium-sized to large domesticated mammals. Other factors are diseases affecting deer, hares and pheasants or their predators; changed hunting pressures on tick hosts or on their predators; provision of winter feed to deer, hares and pheasants; changed agricultural and farming practices; changed grazing methods; changed land use patterns; and reduced or lost diversity of tick host animals and their predators in the "tick's food web" [40,52] including increased abundance or disappearance of vertebrates affecting the abundance or behaviour of blood hosts of ticks; importation and spread of non-native tick host animals; changed environmental and conservation legislation and strategies; creation or establishment of protected or recreational areas such as nature reserves, national parks, forest reserves and urban parks; feeding of deer and other wild animals close to or in urban areas; immigration of deer into urban areas and increased availability for ticks of deer, hares, dogs and pheasants in such areas; greater human exposure to ticks due to changed leisure activities with more outdoor activities to promote well-being and health; increased berry and mushroom picking; more leisure time; changed/increased awareness of and greater ability to detect and remove ticks; increased awareness by doctors and laymen of tick-borne disease symptoms and increased testing for infection leading to increased reporting of ticks bites and tick-borne diseases; greater awareness of "tick high-risk areas" and "TBE high-risk areas"; and changed incidence of TBE due to altered TBE vaccination rates.

### Lyme borreliosis and TBE incidences in Sweden

Roe deer can be infested with *Borrelia- *[53] and presumably also with *Anaplasma-, Rickettsia-, Babesia- *and TBE-virus-infected *I. ricinus*. Therefore, roe deer are likely to play an important role in the dispersal to new locations of ticks infected with human pathogens. Many new TBE-foci have been detected in Sweden during recent years [54-56]. The large roe deer population and the great dispersal potential of deer [16] infested with pathogen-infected ticks, in combination with a warmer climate with more rapid rise in late spring and early summer temperature permitting simultaneous co-feeding of infectible tick larvae together with infected nymphs, may help to explain why such new TBEV-foci have appeared far away from the "old" TBEV-endemic areas. Also, the rapid spread of the tick and tick-borne infections into central and northern Sweden is presumably mainly due to "transportation" of infected ticks on migrating roe deer. It is presumably to a much lesser extent due to dogs, which have visited more southern parts of Sweden, infested with *Borrelia-*infected ticks and northward-migrating birds infested with *Borrelia- *and TBEV-infected ticks [44-46].

In Sweden, the geographical distribution of human-pathogenic LB spirochaetes, *B. burgdorferi *s.l., coincides with that of their main vector, *I. ricinus *[27-30]. In other words, changes in tick distribution and tick density are likely to have increased the risk of human LB and TBE and are likely to lead to further changes in risk areas of these and other tick-borne infections.

The highest incidence of LB reported for Europe is in Eastern Central Europe, with incidence figures of 120-130 human cases per 100,000 inhabitants/year recorded in Slovenia and Austria, respectively [57]. We found that the LB endemic area in Sweden may have a similar, high annual incidence rate: about 125 LB cases/100,000 inhabitants [7]. There are significant relationships between roe deer density and abundance of *I. ricinus *[23,29,30,58], nymphal abundance and density of *Borrelia- *infected nymphs [29,30,39] and between density of *Borrelia- *infected nymphs and LB incidence in humans [57]. Thus, the abundance of roe deer may be used as a crude indicator of risk for human exposure to LB spirochaetes. However, since roe deer are incompetent hosts for *B. burgdorferi *s.l. [53] they may at very high densities divert larval ticks from feeding on reservoir-competent hosts to feeding on deer. This will result in a negative relationship between the density of *I. ricinus *nymphs and the density of nymphs infected with *B. burgdorferi *s.l. [30]. Similar relationships as for LB also exist between roe deer density and tick density and incidence of human TBE cases [59]. Thus, if the abundance and range of roe deer is maintained in Sweden, tick density and the tick's range are likely to increase further. Consequently, the incidences of human diseases vectored by *I. ricinus *are likely to become even higher. The fallow deer, *Dama dama*, occurs in Sweden. It is generally much less abundant than the roe deer and has a lower tendency to disperse compared to that of the roe deer. However, the fallow deer population is on the increase in some localities and is likely to become another important tick host and public health problem, especially in periurban areas in Sweden.

## Conclusions

The results of this follow-up study suggest that *I. ricinus *has continued to expand its range in northern Sweden over the last 16 years where it is now present at up to about 66°N. *Ixodes ricinus *has also become more abundant in Central and Southern Sweden. Since the risk of Lyme borreliosis follows the density of *I. ricinus*, LB is now an emerging risk in North Sweden. Changes in climate, in particular increased duration of the vegetation period and milder winters, and increased abundance of roe deer likely combined to cause the increased range and abundance of the ticks and thereby the risk of tick-borne diseases. The appearance of new TBEV foci may be the result of transportation of TBEV-infected ticks by roe deer and other dispersing or migrating tick hosts such as birds.

The changes in climate that we are observing today are mainly due to human activities [60]. There is an accentuated temperature increase in the Scandinavian mountains, in part due to the tree line advancing into higher altitudes in response to changes in climate [61]. This will increase the potential for the tick to continue to expand its range northwards in Sweden and may also lead to increased transmission of tick-borne pathogens [7,62]. By the end of this century *I. ricinus *infected with *B. burgdorferi *s.l. may, in suitable habitats, include most of the Scandinavian Peninsula apart from the Scandinavian mountain range [7]. Unless populations of large mammalian hosts, in particular deer, of adult ticks are rigorously controlled by hunting and other methods such as protection of lynx and less intensive hunting of the red fox the tick's range and abundance of ticks will most likely increase further. This will presumably lead to increased risk of human tick-borne infections in northern Scandinavia.

## Competing interests

The authors declare that they have no competing interests.

## Authors' contributions

TJ designed the 2009 study, based on the 1994 study, collected and analysed the data, reviewed the literature and wrote the initial and final versions of the manuscript. DJ collected, compiled and co-analysed the data, wrote a computer program and co-refined the intellectual content of the manuscript. LE initiated this research by the 1994 study for which he had the main responsibility; LE and EL co-analysed the data and co-refined the intellectual content of the manuscript. EP co-analysed the data and carried out the statistical analyses. All authors read and approved the final version of the manuscript.

## Author details

TJ and DJ: Medical Entomology Unit, Department of Systematic Biology, Evolutionary Biology Centre, Uppsala University, Norbyvägen 18d, SE-752 36 Uppsala, Sweden. LE, Department of Microbiology, Immunology and Pathology, Colorado State University, Fort Collins, CO 80526, USA. EP: Department of Animal Ecology, Evolutionary Biology Centre, Uppsala University, SE-752 36 Uppsala, Sweden. EL: Institute of Environmental Medicine, Karolinska Institutet, SE-171 77 Stockholm, Sweden.

## Appendix I. Questionnaire used in the 2009 study and published in Swedish in several magazines and newspapers in Sweden

Questionnaire from Uppsala University

Please help us to map the distribution of the tick!

Please answer the questions and send the questionnaire to the address below!

We are very grateful if you would like to answer the following questionnaire.

We are equally interested in 'no' answers as 'yes' answers.

We are particularly interested in data from Norrland (North Sweden) and northern Dalarna. We also want to have any ticks from this area. Place the tick in a jar or box in two well-tied plastic bags. Note that it is important that you fill in your address.

Send the questionnaire (and if possible any ticks) to Thomas Jaenson, EBC, Uppsala University, Norbyvägen 18d, 752 36 Uppsala

Are there ticks within 1 km from your home? YES ____ NO_____ ⁬ ⁬

How many ticks did you find on you, or on your dog, or cat, near/within 1 km from your house in 2008? Please give the total number of ticks found during the whole season per person and per animal

Dog: ______ Cat: ______ Human: ______

Were there any ticks around your home in the early 1990s? YES____ NO______ ⁬ ⁬

Do you think that ticks around your home is less common, about equally common or more common now than in the early 1990s?

Rarer now:____

No change:_____

⁬ More common now: ⁬______

Any other comments of interest for " tick science":

Your name:________________________________

Address:________________________________

Zip Code:_________

Phone number: ______________________

Landskap (province):___________________
